# Transition from Pediatric to Adult Endocrine Care in Patients with Congenital Adrenal Hyperplasia: A Single-Center Retrospective Analysis

**DOI:** 10.3390/medicina62091755

**Published:** 2026-09-11

**Authors:** Filiz Mercan Sarıdaş, Kadircan Karatoprak, Hatice Nursoy, Erhan Hocaoğlu, Müge Yaşar, Yasemin Denkboy Öngen, Soner Cander, Erdinç Ertürk, Özen Öz Gül, Erdal Eren, Canan Ersoy

**Affiliations:** 1 Division of Endocrinology and Metabolism, Department of Internal Medicine, Faculty of Medicine, Bursa Uludağ University, Bursa 16059, Türkiye; 2 Çekirge State Hospital, Bursa 16090, Türkiye; 3 Division of Pediatric Endocrinology and Metabolism, Department of Pediatrics, Faculty of Medicine, Bursa Uludağ University, Bursa 16059, Türkiye; 4 Muş State Hospital, Muş 49200, Türkiye; 5 Şanlıurfa Mehmet Akif İnan Education and Research Hospital, Sanliurfa 63300, Türkiye

**Keywords:** congenital adrenal hyperplasia, transition to adult care, continuity of care, hormonal control, pediatric endocrine care, adult endocrine care

## Abstract

*Background and Objectives*: The transition from pediatric to adult endocrine care represents a vulnerable period for patients with congenital adrenal hyperplasia (CAH), given the risk of loss to follow-up and the evolving physiological and healthcare requirements associated with the transition to adulthood. This study aimed to compare structured and non-structured transition pathways in patients with CAH, focusing on the continuity of adult endocrine care and disease-specific hormonal, clinical, and treatment-related outcomes. *Materials and Methods*: This single-center, retrospective, observational study included patients with CAH who transitioned from pediatric to adult endocrine care and met predefined pediatric and adult follow-up eligibility criteria. Patients were classified according to whether the transition occurred through a structured multidisciplinary transition program or without participation in the structured program. Outcomes were evaluated during predefined observation periods before and after transfer and included continuity-of-care measures, 17-hydroxyprogesterone (17-OHP) and androstenedione concentrations, achievement of predefined hormonal targets, body mass index (BMI), glucocorticoid and mineralocorticoid treatment, and CAH-related clinical outcomes. *Results*: Among 67 patients assessed for eligibility, 29 were included: 20 in the structured transition group and 9 in the non-structured transition group. Overall, 62.1% of the study population was female. No significant between-group differences were observed in continuity-of-care measures, including the pediatric-to-adult care gap, first-year adult visit frequency, maximum visit-free interval, first-year loss to follow-up, or active adult endocrine follow-up at the study cutoff. Pre-transition and post-transfer 17-OHP and androstenedione concentrations and achievement of predefined hormonal targets did not differ significantly between groups. No significant longitudinal changes in hormonal outcomes, BMI, or body-surface-area-adjusted hydrocortisone-equivalent glucocorticoid doses were observed within either group. At the study cutoff, 85.0% of patients in the structured group and 77.8% in the non-structured group remained under active adult endocrine follow-up. *Conclusions*: In this small single-center cohort, no significant differences in the continuity of adult endocrine follow-up or disease-specific outcomes were observed between structured and non-structured transition pathways. Most patients remained engaged with adult endocrine care, and hormonal control showed no evident deterioration following transfer in either group. Given the small sample size and high overall retention, these findings should not be interpreted as evidence of equivalence between the two transition approaches.

## 1. Introduction

Congenital adrenal hyperplasia (CAH) is a chronic, autosomal recessive disorder caused by enzyme deficiencies in the adrenal steroidogenesis pathway and requires lifelong monitoring and treatment. The most common cause is 21-hydroxylase deficiency. Management includes long-term glucocorticoid replacement and, in some cases, mineralocorticoid therapy, as well as monitoring for long-term complications such as short stature, obesity, osteoporosis, and infertility [1,2].

The transition from pediatric to adult endocrine care is a critical and sensitive period in ensuring continuity of care in chronic diseases. During adolescence, hormonal changes, inadequate suppression of adrenal androgens, and psychosocial difficulties may negatively affect treatment adherence and complicate disease management [1,3]. The literature shows that, in chronic diseases requiring lifelong treatment, the transition process is associated with an increased risk of irregular visit attendance, discontinuity of care, and loss to follow-up [4]. CAH-specific studies have similarly shown that the initial transfer to adult services does not necessarily ensure sustained engagement with adult endocrine care [5,6]. Earlier data demonstrated that a substantial proportion of adults with CAH were not receiving specialist adult endocrine follow-up [7], while more recent studies have continued to identify inadequate transition preparation and limited coordination between pediatric and adult services as important barriers to continuity of care [8,9]. Therefore, the transition from childhood to adulthood in CAH should be considered not merely as a transfer of care, but as a continuous healthcare process that requires careful planning.

Structured transition programs incorporating advance preparation, multidisciplinary communication, and coordinated pediatric–adult assessment have therefore been proposed to support continuity of endocrine care [4,9,10]. Current European guidance recommends that transition planning should not be postponed until the final stage of pediatric follow-up, but should instead be initiated during adolescence and, when possible, supported by joint clinical models involving both pediatric and adult care teams [10]. However, evidence regarding the measurable benefit of structured transition models in CAH remains limited. Previous studies have reported variable findings regarding subsequent attendance and retention in adult endocrine care, and loss to follow-up may occur even after apparently successful initial transfer [5,6,11,12].

Importantly, most previous CAH transition studies have focused primarily on transfer completion, clinic attendance, or loss to follow-up, whereas disease-specific outcomes during and after transition have been less extensively evaluated [5,6]. In CAH, continuity of care should ideally be accompanied by maintenance of adequate hormonal control while avoiding both insufficient and excessive glucocorticoid exposure [2,13,14]. Assessment of transition pathways should therefore consider not only engagement with adult endocrine services but also hormonal, treatment-related, and clinical outcomes.

The present study aimed to compare structured and non-structured transition from pediatric to adult endocrine care in patients with CAH, focusing on continuity of adult endocrine follow-up and hormonal, clinical, and treatment-related outcomes before and after transfer.

## 2. Materials and Methods

### 2.1. Study Design

This was a single-center, retrospective, observational study conducted at a tertiary university hospital in Bursa, Türkiye, where pediatric and adult endocrinology outpatient clinics are located within the same hospital. The study included patients diagnosed with CAH who were aged ≥18 years at transfer to adult endocrine care and had at least 12 months of pediatric endocrine follow-up before transfer. To ensure an adequate observation window for first-year outcomes, patients were also required to have at least 12 months of potential follow-up after their first adult endocrinology visit by the study cutoff date (January 2026). Completion of 12 months of actual adult follow-up was not required; therefore, patients who were lost to follow-up during the first year remained eligible for inclusion. Conversely, patients whose first adult endocrinology visit occurred less than 12 months before the study cutoff date were excluded because a complete first-year observation window was not available.

Patients were categorized into two groups according to their transition pathway. The structured transition group comprised patients referred from pediatric to adult endocrine care through the institutional multidisciplinary transition program, which included assessment by the transition council, irrespective of subsequent attendance at the joint pediatric–adult endocrinology clinic. The non-structured transition group comprised patients who met the same eligibility criteria but transferred from pediatric to adult endocrine care during the study period without participation in either the transition council or the joint pediatric–adult endocrinology clinic.

### 2.2. Transition Process

At our center, a structured, multidisciplinary, and protocol-driven transition model has been implemented since December 2015. This model consists of three key components: (1) multidisciplinary case discussions (“transition councils”), (2) a joint outpatient evaluation involving both pediatric and adult endocrinologists, and (3) structured follow-up in adult endocrine care. Transition councils are held regularly and attended by pediatric and adult endocrinologists. During these meetings, the pediatric team presents a comprehensive clinical summary of each patient, and management strategies are jointly reviewed and agreed upon. Patients do not participate in these meetings.

**Figure 1:**
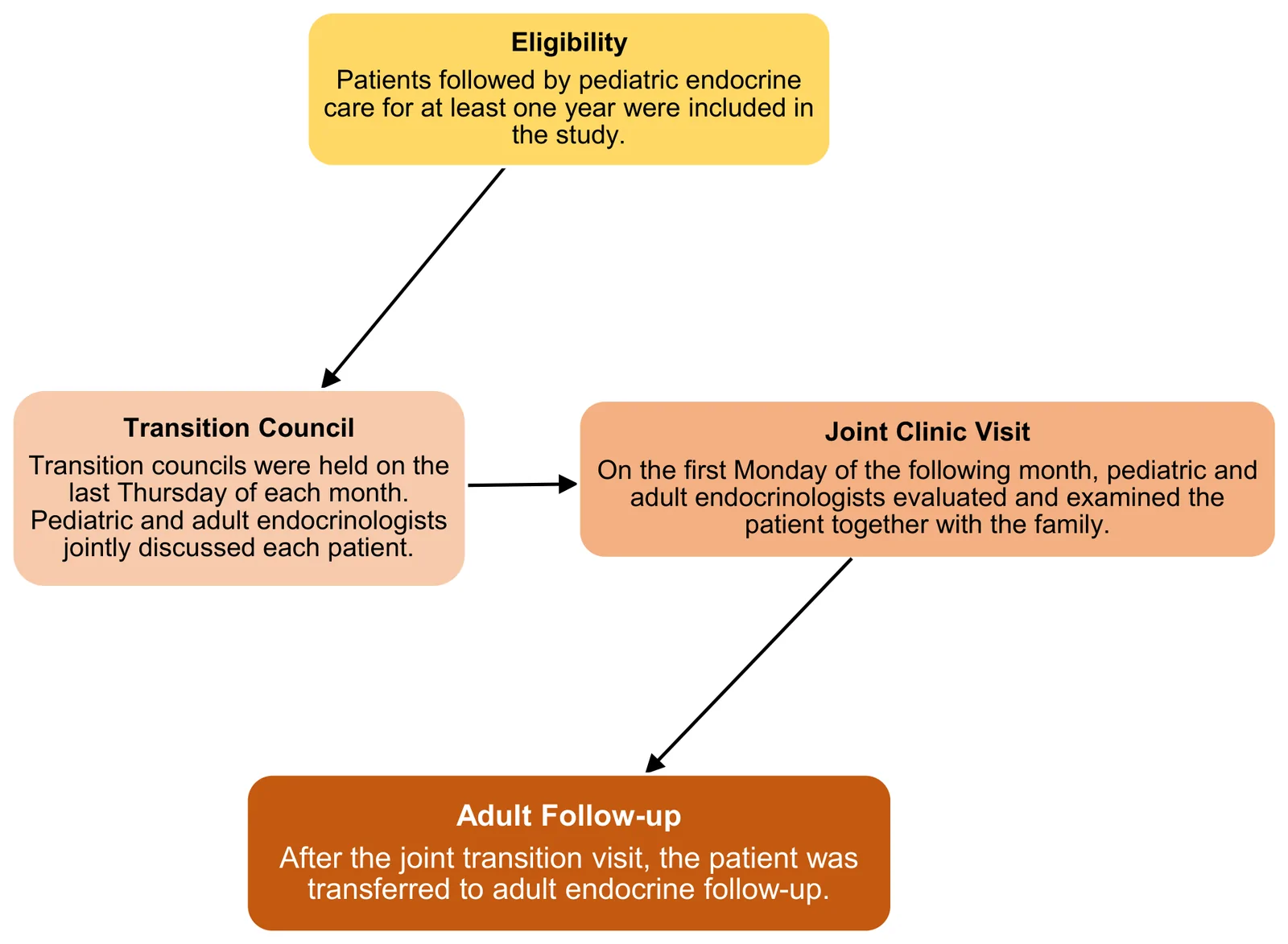
Transition process.

Following the transition council, patients are referred for a joint pediatric–adult endocrinology evaluation, scheduled within the first week. This initial shared evaluation is defined as the joint visit. Subsequent follow-up is continued in the adult endocrinology clinic. The transition process is presented in Figure 1.

### 2.3. Study Assessments

The study outcomes focused on continuity of endocrine care and changes in hormonal, clinical, and treatment-related parameters during the transition from pediatric to adult endocrine care. Continuity-of-care measures included the number of endocrinology visits, the longest interval between consecutive visits, and the care gap between pediatric and adult services. The care gap was defined as the interval between the last pediatric endocrinology visit and the first attended adult endocrinology visit. The maximum visit-free interval during the first adult year was defined as the longest interval without an adult endocrinology visit within 365 days following the first adult endocrinology visit, including the interval between the last visit and the end of the 365-day observation period. Patients who had no adult endocrinology visit or only one visit without continued follow-up during the first post-transition year were considered to be lost to follow-up in the first year. Active adult endocrine follow-up at the study cutoff was defined as having at least one adult endocrinology visit within the 12 months preceding the study cutoff date. Patients without an adult endocrinology visit during this 12-month period were considered to be no longer under active follow-up at the study cutoff.

Hormonal outcomes included 17-hydroxyprogesterone (17-OHP) and androstenedione levels and achievement of predefined hormonal targets. Outcomes were evaluated across three predefined observation periods. The pre-transition period was defined as the 12 months preceding the last pediatric endocrinology visit before transfer to adult care. The first adult year was defined as the 12 months following the first attended adult endocrinology visit. For patients with more than 2 years of adult endocrine follow-up, the last follow-up period was defined as the most recent 12 months of adult endocrine care.

### 2.4. Data Collection

Demographic and clinical data were retrospectively extracted from electronic medical records. Variables included age, sex, age at CAH diagnosis, CAH phenotype, duration of pediatric endocrine follow-up, age at the first adult endocrinology visit, and duration of adult endocrine follow-up. 

The number of endocrinology visits and the longest interval between consecutive visits were recorded for each observation period. Repeated visits related to the same event occurring within 10 days were counted as a single visit.

Treatment-related variables included glucocorticoid type, daily glucocorticoid dose, body-surface-area-adjusted hydrocortisone-equivalent dose, mineralocorticoid use and dose. Body surface area was calculated using the Mosteller formula. Glucocorticoid doses were converted to hydrocortisone-equivalent doses using standard glucocorticoid potency equivalences and were adjusted for body surface area.

Available genetic testing results were retrospectively retrieved from the medical records. Genetic testing was not performed uniformly across the study population. Results were classified according to whether a pathogenic or likely pathogenic CAH-related variant was identified; inconclusive results were recorded separately. Genetic findings were analyzed descriptively.

### 2.5. Hormonal and Clinical Assessment

The principal hormonal parameters were 17-OHP and androstenedione. For these parameters, the median of all available measurements within each study period was calculated to reduce measurement variability. For study purposes, predefined hormonal targets were a period median 17-OHP level ≤ 12 ng/mL and a period median androstenedione level within the age- and sex-specific reference range [13,15,16]. Combined target achievement was defined as fulfillment of both criteria within the same observation period. Patients with a missing value for either parameter were excluded from the combined target analysis.

Clinical assessment included body mass index (BMI), adrenal crisis, adrenal crisis-related emergency department visits and hospitalizations, menstrual regularity in females, and testicular adrenal rest tumor (TART) in males. Menstrual cycles were classified as regular or irregular. TART was assessed according to ultrasonographic findings. Menstrual regularity and TART were analyzed only in patients with available assessments during the respective observation period.

### 2.6. Statistical Analysis

The distribution of continuous variables was assessed using the Shapiro–Wilk test. Continuous variables were expressed as mean ± standard deviation or median (IQR; 25th–75th percentiles), as appropriate, and categorical variables as number and percentage. Given the small sample size and distributional characteristics of the data, non-parametric methods were used for the principal comparative analyses. Between-group comparisons of continuous variables were performed using the Mann–Whitney U test, whereas categorical variables were compared using Fisher’s exact test or an exact test appropriate to the dimensions of the contingency table. Changes in continuous variables across the three observation periods were evaluated separately within each transition group using the Friedman test. Changes in repeated binary categorical variables across three observation periods were assessed using Cochran’s Q test. Repeated-measures analyses were restricted to patients with available data at all time points included in the respective analysis. For cross-sectional comparisons at each observation period, all available data at that time point were used. Missing data were not imputed, and denominators were reported where the number of available observations differed from the total group size. Exact two-sided *p* values were used where appropriate because of the small sample size and sparse contingency tables. A two-sided *p* value < 0.05 was considered significant. As an exploratory analysis, early continuity-of-care measures were compared between patients who remained under active adult endocrine follow-up at the study cutoff and those who did not. Continuous variables were compared using the Mann–Whitney U test, with exact *p* values reported because of the small number of patients no longer under active follow-up. Statistical analyses were performed using IBM SPSS Statistics for Macintosh, version 29.0 (IBM Corp., Armonk, NY, USA).

### 2.7. Ethics

The study was approved by the Bursa Uludağ University Health Research Ethics Committee on 21 January 2026 (approval number: 2026/55/2-29) and was conducted in accordance with the Declaration of Helsinki.

## 3. Results

### 3.1. Pre-Transition Patient Characteristics

**Figure 2:**
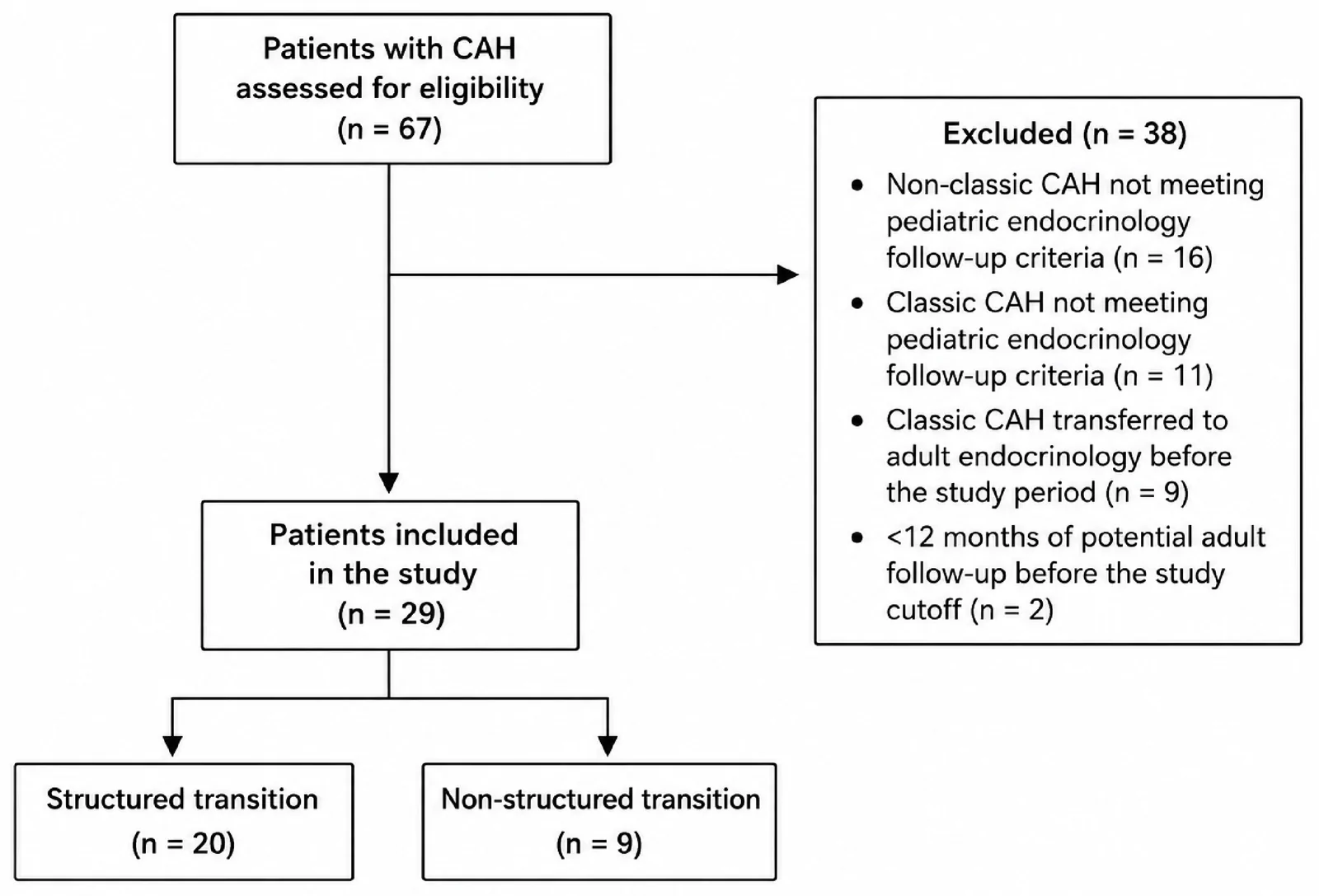
Flow diagram of patient selection and classification into structured and non-structured transition groups.

A total of 67 patients with CAH were assessed for eligibility. Of these, 38 were excluded, resulting in a final study cohort of 29 patients, including 20 in the structured transition group and 9 in the non-structured transition group (Figure 2). Overall, 62.1% of the study population was female.

The pre-transition characteristics are summarized in Table 1. No significant between-group differences were observed in demographic, clinical, hormonal, or treatment-related characteristics. The median age at the first adult endocrinology visit was 19 years in both groups, while the median duration of pediatric endocrine follow-up was 17.11 years in the structured transition group and 12.96 years in the non-structured transition group (*p* = 0.253). Median pre-transition 17-OHP [8.60 vs. 18.14 ng/mL; *p* = 0.594] and androstenedione concentrations [1.63 vs. 4.09 mcg/L; *p* = 0.108] did not differ significantly between the structured and non-structured transition groups.

Genetic testing was performed in 19 of 29 patients (65.5%). Pathogenic or likely pathogenic CAH-related variants were documented in 16 patients, predominantly involving CYP21A2. One genetic analysis was inconclusive, while no pathogenic variant was identified in two of the tested patients. Because genetic testing was not performed uniformly and the tested genes and methods varied according to clinical indication and time period, genetic findings were reported descriptively.

### 3.2. Follow-Up Continuity and Transition Outcomes

No significant differences in continuity-of-care measures were observed between the structured and non-structured transition groups (Table 2), including the pediatric-to-adult care gap, number of adult endocrinology visits during the first adult year, maximum visit-free interval, number of visits during the most recent 12-month follow-up period, loss to follow-up during the first adult year, active adult endocrine follow-up at the study cutoff, or duration of adult endocrine follow-up.

In an exploratory analysis of the overall cohort, the number of adult endocrinology visits during the first adult year and the pediatric-to-adult care gap did not differ between patients who remained under active adult endocrine follow-up at the study cutoff and those who did not (exact *p* = 0.978 and *p* = 0.845, respectively). The maximum visit-free interval during the first adult year was longer among patients who were no longer under active follow-up [244.0 (155.5–315.5) vs. 164.0 (102.75–195.75) days], although the difference was not significant (exact *p* = 0.078). Given the small number of patients who were no longer under active follow-up (*n* = 5), these findings should be considered exploratory.

### 3.3. Hormonal, Anthropometric, and Treatment-Related Outcomes During Follow-Up

No significant between-group differences in hormonal outcomes were observed across the evaluated time points (Table 3). Median 17-OHP and androstenedione concentrations and the proportions of patients meeting their predefined targets did not differ significantly between groups at the pre-transition assessment, during the first adult year, or at last follow-up. Similarly, achievement of both predefined hormonal targets did not differ significantly between groups at any time point. Among patients with complete data across all three observation periods, no significant longitudinal changes in these outcomes were observed within either transition group.

BMI did not differ significantly between the structured and non-structured transition groups at any of the three observation periods (Table 3). In the structured transition group, BMI increased numerically from a median of 25.65 kg/m^2^ pre-transition to 27.56 kg/m^2^ at last follow-up, although the longitudinal change was not significant (*p* = 0.091). No significant longitudinal change was observed in the non-structured group (*p* = 0.259).

No significant between-group differences in body-surface-area-adjusted hydrocortisone-equivalent glucocorticoid doses were observed at any of the three time points, although the pre-transition dose was numerically higher in the non-structured transition group [11.69 (8.91–16.14) vs. 9.35 (5.91–11.76) mg/m^2^/day; *p* = 0.077]. No significant longitudinal changes in glucocorticoid doses were observed within either group. Mineralocorticoid use and doses also showed no significant between-group or longitudinal differences.

No significant between-group differences in menstrual irregularity or TART occurrence were observed at the available time points. No significant longitudinal change in menstrual irregularity was observed in the structured transition group (*p* = 0.667); longitudinal analysis was not performed in the non-structured group because of the limited number of complete observations. TART occurrence did not differ significantly between the groups at any of the evaluated time points; longitudinal analysis was not performed because of the limited number of patients with complete assessments across all three time points.

No adrenal crisis-related emergency department visits or hospitalizations were recorded in either group during the pre-transition period or the first adult year. At last follow-up, two patients in the structured transition group had adrenal crisis-related emergency department visits, one of whom required hospitalization. No adrenal crisis-related events were recorded in the non-structured transition group at last follow-up. Given the small number of events, these outcomes were reported descriptively.

### 3.4. Glucocorticoid Treatment Patterns

Glucocorticoid treatment patterns over follow-up are presented in Table 4. Dexamethasone was the most frequently used regimen during the pre-transition period in both groups. During adult follow-up, the proportion of patients receiving dexamethasone monotherapy decreased, while greater variation in glucocorticoid regimens was observed, particularly in the structured transition group.

## 4. Discussion

In this single-center retrospective study of patients with CAH transitioning from pediatric to adult endocrine care, no significant differences were observed between structured and non-structured transition pathways in continuity of adult endocrine follow-up or disease-specific outcomes. No significant between-group differences were identified in the pediatric-to-adult care gap, number of adult endocrinology visits during the first adult year, maximum visit-free interval, retention in active adult endocrine follow-up, or duration of adult follow-up. Similarly, no significant between-group differences were observed in hormonal outcomes, including 17-OHP and androstenedione concentrations and achievement of the predefined hormonal targets. Furthermore, among patients with sufficient longitudinal data, hormonal and treatment-related remained generally stable over time. Overall, continuity of adult endocrine care was maintained in most patients; however, given the small sample size and high overall retention, the absence of significant between-group differences should not be interpreted as evidence of equivalence between the two transition pathways.

Previous studies in patients with CAH have shown that initial transfer to adult endocrine care does not necessarily ensure sustained engagement during subsequent follow-up. In their 18-year single-center study, Gleeson et al. highlighted the challenges of maintaining long-term endocrine follow-up in patients with CAH and reported that patients with more regular and frequent pediatric endocrinology visits before transition were more likely to remain engaged with endocrine care during adulthood [5]. More recently, a study evaluating a joint transition model integrating pediatric and adult endocrine services reported a high rate of initial transfer to adult care; however, a substantial proportion of patients subsequently disengaged from adult endocrine follow-up [6].

In the present study, no significant differences were observed between structured and non-structured transition pathways in continuity-of-care measures. Loss to follow-up during the first adult year occurred in 15.0% of the structured group and in none of the non-structured group, whereas 85.0% and 77.8%, respectively, remained under active adult endocrine follow-up at the study cutoff; neither difference was significant. These findings should not be interpreted as indicating equivalence between the two approaches or as evidence against structured transition. The small sample size and the limited number of loss-to-follow-up events limited the statistical power to detect differences between the two transition pathways. Moreover, pediatric and adult endocrine services were located within the same tertiary-care institution, which may itself facilitate continuity even among patients who did not undergo the formal transition program.

Loss to follow-up remains a major challenge in the transition from pediatric to adult care with considerable variability reported across studies because of differences in patient populations, healthcare systems, follow-up duration, and definitions of loss to follow-up [5,6,12,17,18,19]. Disengagement may also be influenced by factors beyond the transition pathway itself, including geographical distance, missed appointments, socioeconomic factors, and other logistical barriers [5,12,17]. In our cohort, some patients who disengaged from follow-up had identifiable circumstances that could interfere with continuity of care, including residence outside the city or relocation abroad. Therefore, absence from follow-up at our center may not invariably represent complete disengagement from endocrine care, as follow-up at another institution could not always be fully ascertained. Notably, one patient who was initially classified as lost to follow-up re-entered adult care 1.5 years later and remained under follow-up thereafter, suggesting that disengagement from care may, in some cases, be temporary rather than permanent.

An exploratory observation concerned early patterns of engagement with adult endocrine care. Patients who were no longer under active adult endocrine follow-up at the study cutoff had a numerically longer maximum visit-free interval during the first adult year than those who remained under active follow-up [244.0 (155.5–315.5) vs. 164.0 (102.75–195.75) days], although this difference was not significant (exact *p* = 0.078). In contrast, the number of adult endocrinology visits during the first year and the pediatric-to-adult care gap did not differ according to subsequent follow-up status. Given that only five patients were no longer under active follow-up, this analysis should be considered hypothesis-generating rather than confirmatory.

Nevertheless, this observation is consistent with previous reports emphasizing the importance of patterns of healthcare engagement around the time of transfer. Downing et al. reported that patients with poor visit attendance before transfer also demonstrated poor attendance after transfer [20]. Similarly, Davidse et al. [12] noted that patients with a greater number of missed appointments were more likely to discontinue follow-up. The current ESPE/ESE transition guideline likewise recommends scheduling the first adult care appointment within 3–6 months to minimize gaps in care during the transition process [10]. Collectively, these findings suggest that continuity of care may be better conceptualized as a longitudinal pattern of healthcare engagement rather than as the success or failure of a single transfer event. Prolonged gaps between visits early after transfer may therefore warrant particular attention as a potential signal for patients requiring additional support, although this hypothesis requires confirmation in larger cohorts.

A central aspect of the present study was the evaluation of disease-specific hormonal outcomes in addition to healthcare attendance. No significant between-group differences were observed in median 17-OHP and androstenedione concentrations or in the proportions of patients meeting the predefined hormonal targets at the evaluated time points. Moreover, no significant longitudinal changes in these parameters were observed among patients with complete follow-up data. Taken together, these findings suggest that hormonal control did not show evident deterioration following transfer to adult endocrine care in either transition pathway.

This observation is clinically relevant because adolescence and young adulthood represent periods during which treatment adherence and optimization of glucocorticoid replacement may be challenging [1,3,14]. Monitoring in CAH requires balancing suppression of excess adrenal androgen production against the adverse consequences of excessive glucocorticoid exposure, and 17-OHP and androstenedione remain important markers of disease monitoring when interpreted in the appropriate clinical and sampling context [2,13]. The absence of significant differences between the two transition pathways should, however, be interpreted cautiously given the small sample size and the multifactorial nature of hormonal control in CAH.

Glucocorticoid treatment patterns became more heterogeneous after transfer to adult care, while no significant longitudinal change was observed in body-surface-area-adjusted hydrocortisone-equivalent doses. Dexamethasone use decreased during adult follow-up, accompanied by greater variation in glucocorticoid regimens. These changes may reflect individualization of treatment according to clinical circumstances rather than a systematic change in overall glucocorticoid exposure. This observation is relevant given the need in adult CAH management to balance adequate androgen suppression against the potential long-term consequences of excessive glucocorticoid exposure [2,13,21].

No clear longitudinal changes were observed in the evaluated clinical outcomes, including menstrual irregularity and testicular adrenal rest tumor occurrence, although these assessments were limited by the small number of patients with available data. Body mass index increased numerically over time in the structured transition group, although the longitudinal change was not significant. The present study does not allow conclusions regarding the relationship between glucocorticoid treatment and changes in BMI. Nevertheless, body weight in adults with CAH may be influenced by multiple factors, and continued cardiometabolic surveillance remains important given the increased adiposity and adverse metabolic outcomes reported in previous cohorts [21,22,23].

A major strength of this study is the inclusion of a non-structured transition comparison group from the same center, allowing structured and non-structured transition pathways to be evaluated within the same healthcare setting. The study also provides real-world longitudinal data on a rare disease population over nearly a decade of experience at a tertiary referral center. Importantly, transition was evaluated as a longitudinal process rather than solely as completion of transfer, incorporating continuity of adult endocrine follow-up together with hormonal, treatment-related, and clinical outcomes. The use of predefined observation periods before transfer, during the first adult year, and during subsequent follow-up allowed these outcomes to be evaluated across the different stages of the transition. Given the limited data on the transition from pediatric to adult endocrine care in patients with CAH in Türkiye, this study contributes real-world evidence to the existing literature.

Several limitations should be acknowledged. First, the retrospective, single-center design and small sample size, particularly in the non-structured transition group, limited the statistical power to detect differences between the transition pathways. Therefore, the absence of statistical significance between-group differences should not be interpreted as evidence of equivalence between structured and non-structured transition. Second, some hormonal, and clinical variables were unavailable at all observation periods, resulting in smaller sample sizes for longitudinal analyses, particularly in the non-structured group, and limiting longitudinal assessment of some outcomes. Third, hormone measurements were obtained during routine clinical practice rather than according to a standardized prospective sampling protocol; therefore, variability related to sampling conditions cannot be excluded. Fourth, the small number of patients who were no longer under active adult endocrine follow-up limited comparisons according to follow-up status; therefore, these findings should be considered exploratory. Finally, because both pediatric and adult endocrine services were provided within the same tertiary-care institution, the findings may not be directly generalizable to healthcare systems in which transition requires transfer between different institutions or geographic locations. In addition, follow-up at other institutions could not always be fully ascertained; therefore, absence from follow-up at our center may not necessarily indicate complete disengagement from endocrine care.

## 5. Conclusions

In conclusion, no significant differences in continuity of adult endocrine follow-up or disease-specific outcomes were observed between structured and non-structured transition pathways in this small single-center cohort of patients with CAH. Most patients remained engaged with adult endocrine care, and no evident deterioration in hormonal control was observed following transfer in either transition pathway. Given the small sample size and high overall retention in both groups, these findings should not be interpreted as evidence of equivalence between the two transition approaches. Future studies should focus on identifying which aspects of the transition process are associated with sustained engagement in adult endocrine care while maintaining adequate disease control in patients with CAH.

## Figures and Tables

**Table 1:** Pre-transition clinical, hormonal, and treatment characteristics of the structured and non-structured transition groups.

	Structured Transition (*n* = 20) Median (Q1–Q3) or *n* (%)	Non-Structured Transition (*n* = 9) Median (Q1–Q3) or *n* (%)	*p*-Value
Female sex	13 (65)	5 (55.6)	0.694
Age at first diagnosis, years	2 (0–5)	0 (0–2.25)	0.119
Age at first adult endocrinology visit, years	19 (18–20)	19 (18–20.5)	0.927
CAH etiology			
21-hydroxylase deficiency	18 (90)	9 (100)	
11β-hydroxylase deficiency	2 (10)		
Phenotype among patients with 21-hydroxylase deficiency, *n* (%)			
Salt-wasting	10 (55.6)	7 (77.8)	
Simple virilizing	7 (38.9)	2 (22.2)	
Non-classic	1 (5.6)	-	
Duration of pediatric endocrine follow-up, years	17.11 (12.01–18.46)	12.96 (9.03–18)	0.253
Pediatric endocrinology visits during the last year of pediatric care	5 (3.25–6)	5 (2.5–6.5)	0.839
BMI at the last pediatric visit, kg/m^2^	25.65 (21.67–28.45)	25.04 (21.88–27.62)	0.627
BMI category			
Normal	9 (45)	4 (44.4)	0.412
Overweight	7 (35)	5 (55.6)	
Obese	4 (20)	0 (0.00)	
17-OHP, ng/mL	8.6 (2.70–42.21)	18.14 (3.58–34.58)	0.594
17-OHP ≤ 12 ng/mL	11 (55)	4 (44.4)	0.700
Androstenedione mcg/L	1.63 (0.83–4.58)	4.09 (1.68–8.94) (*n* = 8)	0.108
Androstenedione within age- and sex-specific reference range	14 (70)	3 (37.5)	0.200
Both hormonal targets achieved	11/20 (55)	2/8 (25)	0.221
Glucocorticoid type			
Hydrocortisone	6 (30)	3 (33.3)	0.445
Dexamethasone	14 (70)	5 (55.6)	
Deflazacort	0 (0)	1 (11.1)	
Hydrocortisone-equivalent glucocorticoid dose, mg/m^2^/day	9.35 (5.91–11.76)	11.69 (8.91–16.14)	0.077
Mineralocorticoid use	8 (40)	4 (44.4)	1.000
Genetic testing available, *n/N* (%)	16/20 (80.0)	3/9 (33.3)	
Pathogenic/likely pathogenic CAH-related variant identified, *n/N (*%)	14/20 (70)	2/9 (22.2)	

Between-group comparisons were performed using the Mann–Whitney U test for continuous variables and Fisher’s exact test for categorical variables, as appropriate. For categorical variables presented as *n/N* (%), *n* indicates the number of patients with the specified characteristic and *N* indicates the total number of patients with available data for the corresponding variable. Percentages were calculated based on available data; therefore, denominators may vary because of missing data. Genetic testing was not performed uniformly across the study population; therefore, genetic findings were reported descriptively without between-group statistical comparison. 17-OHP, 17-hydroxyprogesterone; BMI, body mass index; CAH, congenital adrenal hyperplasia.

**Table 2:** Continuity of adult endocrine care after transition.

	Structured Transition (*n* = 20) Median (Q1–Q3) or *n* (%)	Non-Structured Transition (*n* = 9) Median (Q1–Q3) or *n* (%)	*p*-Value
Pediatric to adult care gap, days	65 (19.5–164)	122 (24.5–281)	0.495
Adult endocrinology visits during the first adult year	4 (2.25–6)	6 (4–8)	0.093
Maximum visit-free interval during the first year, days	173.0 (105.25–238.50)	179.0 (114.50–216.50)	0.835
Adult endocrinology visits during the most recent 12-month follow-up period	5 (2.25–6), *n* = 16	6 (3–6), *n* = 7	0.710
Loss to follow-up during the first adult year	3 (15)	0	0.532
Active adult endocrinology follow-up at study cut off	17 (85)	7 (77.8)	0.633
Duration of adult endocrine follow-up, years	4.27 (2.14–5.79)	4.21 (2.90–7.79)	0.411

**Table 3:** Longitudinal anthropometric, hormonal, and treatment- outcomes in the structured and non-structured transition groups.

	Pre-Transition	First Adult Year	Last Follow-Up	Within Group *p*-Value
17-OHP, ng/mL, median (Q1–Q3)				
Structured transition	8.6 (2.70–42.21), *n* = 20	6.14 (1.82–76.60), *n* = 17	15.45 (2.87–75.53), *n* = 16	0.982, (*n* = 15)
Non-structured transition	18.14 (3.58–34.58), *n* = 9	11.00 (2.66–24.13), *n* = 9	4.07 (2.72–19.50), *n* = 7	1.000, (*n* = 7)
Between group *p*-value	0.594	0.833	0.452	
17-OHP ≤ 12 ng/mL, n/N (%)				
Structured transition	11/20 (55)	9/17 (52.9)	7/16 (43.8)	0.486 (*n* = 15)
Non-structured transition	4/9 (44.4)	5/9 (55.6)	5/7 (71.4)	0.667
Between group *p*-value	0.700	1.000	0.371	
Androstenedione, median (Q1–Q3)				
Structured transition	1.63 (0.83–4.58), *n* = 20	1.85 (0.30–4.07), *n* = 17	1.15 (0.30–3.60), *n* = 15	0.527, *n* = 14
Non-structured transition	4.09 (1.68–8.94), *n* = 8	1.93 (0.43–16.35), *n* = 8	0.30 (0.30–15.43), *n* = 5	0.778, *n* = 3
Between group *p*-value	0.108	0.511	0.655	
Androstenedione within age- and sex-specific reference range, *n* (%)				
Structured transition	14/20 (70)	11/17 (64.7)	11/15 (73.3)	1.000, *n* = 14
Non-structured transition	3/8 (37.5)	6/8 (75)	4/5 (80)	1.000 (*n* = 3)
Between group *p*-value	0.200	1.000	1.000	
Achievement of both predefined hormonal targets, *n* (%)				
Structured transition	11/20 (55)	9/17 (52.9)	7/15 (46.7)	0.486, *n* = 14
Non-structured transition	2/8 (25)	4/8 (50)	3/5 (60)	1.000 (*n* = 3)
Between group *p*-value	0.221	1.000	1.000	
BMI, kg/m^2^, median (IQR)				
Structured transition	25.65 (21.67–28.45), *n* = 20	26.22 (22.46–30.37), *n* = 17	27.56 (23.04–32.32), *n* = 16	0.091, *n* = 15
Non-structured transition	25.04 (21.88–27.62), *n* = 9	23.83 (22.47–27.90), *n =* 9	25.39 (23.42–28.16), *n* = 7	0.259, *n* = 7
Between group *p*-value	0.627	0.525	0.413	
Glucocorticoid dose, HC-equivalent, mg/m^2^/day				
Structured transition	9.35 (5.91–11.76), *n* = 20	10.10 (6.70–11.98), *n* = 17	10.99 (8.46–15.75), *n* = 16	0.291
Non-structured transition	11.69 (8.91–16.14), *n* = 9	10.95 (8.66–14.37), *n* = 9	12.73 (7.73–16.43), *n* = 7	0.470
Between group *p*-value	0.077	0.367	1.000	
Mineralocorticoid use, *n* (%)				
Structured transition	8/20 (40)	7/17 (41.2)	6/16 (37.5)	
Non-structured transition	4/9 (44.4)	4/9 (44.4)	4/7 (57.1)	
Between group *p*-value	1.000	1.000	0.650	
Mineralocorticoid dose				
Structured transition	0.100 (0.081–0.181), *n* = 8	0.100 (0.050–0.200), *n* = 7	0.100 (0.088–0.125), *n* = 6	0.667 (*n* = 6)
Non-structured transition	0.100 (0.100–0.175), *n* = 4	0.100 (0.100–0.175), *n* = 4	0.100 (0.100–0.175), *n* = 4	1.000 (*n* = 4)
Between group *p*-value	0.917	0.473	0.633	
Menstrual irregularity, *n/N* (%)				
Structured transition	0/12 (0)	2/12 (16.7)	2/10 (20)	0.667 †
Non-structured transition	1/5 (20)	0/4 (0)	0/2 (0)	NA
Between group *p*-value	0.294	1.000	1.000	
Testicular adrenal rest tumor (TART), *n/N* (%)				
Structured transition	4/7 (57.1)	4/7 (57.1)	4/6 (66.7%)	
Non-structured transition	3/3 (100)	3/3 (100)	4/4 (100)	
Between group *p*-value	0.475	0.475	0.467	

Data are presented as median (Q1–Q3) or *n/N* (%), as appropriate. For categorical outcomes presented as *n/N* (%), *n* indicates the number of patients meeting the specified criterion, and *N* indicates the total number of patients with available data for that outcome at the corresponding time point. Between-group comparisons at each time point were performed using the Mann–Whitney U test for continuous variables and Fisher’s exact test for categorical variables. Within-group changes across the three time points were assessed using the Friedman test for continuous variables and Cochran’s Q test for categorical variables. Longitudinal analyses were restricted to patients with available data at all three time points for the respective outcome. Exact *p*-values are reported for within-group analyses because of the small sample size. Missing data were not imputed. A two-sided *p*-value < 0.05 was considered significant. Achievement of both predefined hormonal targets was defined as a 17-OHP level ≤ 12 ng/mL and an androstenedione level within the age- and sex-specific reference range. Patients with missing data for either hormonal parameter were excluded from the composite outcome analysis. † Cochran’s Q exact test, based on 10 patients with complete data at all three time points. Longitudinal analysis was not performed in the non-structured transition group because of an insufficient number of complete observations. Abbreviations: 17-OHP, 17-hydroxyprogesterone; BMI, body mass index; HC, hydrocortisone; IQR, interquartile range; NA, not applicable; TART, testicular adrenal rest tumor.

**Table 4:** Glucocorticoid regimens during transition to adult endocrine care.

Glucocorticoid Regimen, *n/N* (%)	Structured Transition			Non-Structured Transition		
	Pre-Transition	First Adult Year	Last Follow-Up	Pre-Transition	First Adult Year	Last Follow-Up
Dexamethasone	14/20 (70.0)	10/17 (58.8)	5/16 (31.3)	5/9 (55.6)	6/9 (66.7)	3/7 (42.9)
Hydrocortisone	6/20 (30.0)	5/17 (29.4)	4/16 (25.0)	3/9 (33.3)	2/9 (22.2)	1/7 (14.3)
Prednisolone	0/20 (0.0)	1/17 (5.9)	2/16 (12.5)	0/9 (0.0)	0/9 (0.0)	1/7 (14.3)
Deflazacort	0/20 (0.0)	0/17 (0.0)	0/16 (0.0)	1/9 (11.1)	1/9 (11.1)	1/7 (14.3)
Prednisolone + hydrocortisone	0/20 (0.0)	1/17 (5.9)	2/16 (12.5)	0/9 (0.0)	0/9 (0.0)	0/7 (0.0)
Dexamethasone + prednisolone	0/20 (0.0)	0/17 (0.0)	2/16 (12.5)	0/9 (0.0)	0/9 (0.0)	0/7 (0.0)
Dexamethasone + hydrocortisone	0/20 (0.0)	0/17 (0.0)	1/16 (6.3)	0/9 (0.0)	0/9 (0.0)	1/7 (14.3)

Data are presented as *n/N* (%). Denominators vary according to available follow-up data at each time point. Each patient was classified according to the glucocorticoid regimen used at the respective time point, and combination regimens were considered separate treatment categories.

## Data Availability

The data that support the findings of this study are available from the corresponding author upon reasonable request. The data are not publicly available due to patient privacy and confidentiality considerations.
